# Aerobic Isolates from Gestational and Non-Gestational Lactating Bitches (*Canis lupus familiaris*)

**DOI:** 10.3390/ani11113259

**Published:** 2021-11-14

**Authors:** Iosif Vasiu, Gabriele Meroni, Roman Dąbrowski, Piera Anna Martino, Asta Tvarijonaviciute, Mariola Bochniarz, Raul Alexandru Pop, Florinel Gheorghe Brudaşcă, Nicodim Iosif Fiţ

**Affiliations:** 1 Department and Clinic of Infectious Disease, Faculty of Veterinary Medicine, University of Agricultural Science and Veterinary Medicine, 400372 Cluj-Napoca, Romania; iosif.vasiu@usamvcluj.ro (I.V.); florinel.brudasca@usamvcluj.ro (F.G.B.); 2 One Health Unit, Department of Biomedical Surgical and Dental Sciences, School of Medicine, University of Milan, 20133 Milan, Italy; gabriele.meroni@unimi.it (G.M.); piera.martino@unimi.it (P.A.M.); 3 Department and Clinic of Animal Reproduction, Faculty of Veterinary Medicine, University of Life Sciences, 20-612 Lublin, Poland; mariolabochniarz@interia.pl; 4 Interdisciplinary Laboratory of Clinical Analysis, Interlab-UMU, Regional Campus of International Excellence “Mare Nostrum”, University of Murcia, 30100 Murcia, Spain; asta@um.es; 5 Department of Obstetrics and Gynecology, Faculty of Veterinary Medicine, University of Agricultural Science and Veterinary Medicine, 400372 Cluj-Napoca, Romania; quantasbasset@yahoo.com; 6 Department of Microbiology, Mycology, and Immunology, Faculty of Veterinary Medicine, University of Agricultural Science and Veterinary Medicine, 400372 Cluj-Napoca, Romania; nfit@usamvcluj.ro

**Keywords:** dog (*Canis lupus familiaris*), lactation, mastitis, bacterial etiology

## Abstract

**Simple Summary:**

Mastitis represents the inflammation of the mammary gland, and it affects all mammals. It is usually caused by bacterial agents, but other organisms such as fungi or parasites can be responsible for the onset of mastitis. In bitches, mastitis is considered a genuine emergency, since it can affect both the mother and the pups. Left untreated, it can lead to the loss of the bitch and the entire litter. The aim of this study was to evaluate the bacterial load from the milk of healthy bitches and from bitches with mastitis. The main isolated bacterial families were the Staphylococcaceae, Enterobacteriaceae and Enterococcaceae families. The bacterial load from bitches’ overt pseudopregnancy seems to have a lower bacteriological burden than periparturient females. Some of the isolated milk strains of lactating bitches are also responsible for nosocomial infection. The transmission of such strains from humans to animals or vice versa is possible. Environmental hygiene needs to be adequately addressed, alongside a minimum manipulation of the lactating mammary glands where possible.

**Abstract:**

Mastitis is a complex and well-defined mammary gland pathology, and an emergency in bitches. In dogs, its prevalence is about 1% of all reported diseases and about 5.3% of all reproductive pathologies. Lactating bitches are naturally prone to developing mastitis since puppies can easily overstimulate the epidermal layer of nipples during feeding, facilitating bacterial colonization of the glands. This study aimed to describe the aerobic bacterial flora isolated from milk samples derived from a cohort of patients (*n* = 87) diagnosed with clinical mastitis (*n* = 29), subclinical mastitis (*n* = 17) and healthy mammary glands (*n* = 46). All of the patients underwent a gynecology consultation to diagnose mammary gland afflictions; physical examination results were coupled with traditional hematological findings. The milk samples were plated on specific microbiological media for bacterial isolation. Among the 162 milk samples analyzed, 93.2% (151/162) had a positive microbiological result, while 6.8% (11/162) were sterile. The bacteriological profile of the milk samples showed 47 different species. The most common bacterial families detected in healthy bitches and bitches with subclinical and clinical mastitis were the Staphylococcaceae, Enterobacteriaceae and Enterococcaceae families. The results indicated that half of the isolated bacteria are novel findings in dogs and that some of them are normal components of human milk.

## 1. Introduction

In bitches, mastitis, the inflammation of the mammary gland [1,2], usually has a bacterial origin [3,4], but mycotic [5] and parasitical etiology [6,7,8] has also been reported. Most often, mastitis appears during the post-partum period, but it has also been signaled in bitches with lactomania (*Lactatio sine graviditate*, *graviditas nervosa*, overt pseudopregnancy) [9] and can also be diagnosed in females that suffer from different systemic diseases, such as diabetes mellitus [10].

Two routes of mammary gland infection have been proposed. The ascending route is the most common means of infection. During lactation, puppies can promote the entry of pathogens through the lesions they cause with teeth and nails (mastitis caused by *Staphylococcus aureus* or *Streptococcus agalactiae*) or environmental factors such as the use of improper bedding (*Escherichia coli*), poor hygiene or even self-licking by the bitch, especially when the mammary glands are not completely empty (small litters, premature weaning, overt pseudopregnancy) [3,9,11,12,13,14]. The descendent route is responsible for infections that appear in the post-partum period and are usually secondary to metritis [15,16] and vaginitis [17]. Finally, infections of the *mammae* are favored by local vessel development [3], which causes bacterial emboli, affecting the most developed and irrigated mammary glands in the periparturient period [15].

Bitches suffering from mastitis can be asymptomatic or present local (discolored milk and hot, red, engorged and painful mammary glands) and general signs of illness (depression, lethargy, vomiting, dehydration, anorexia and fever) [18,19]. However, severe cases of mastitis can be complicated by the animal’s septic shock, death [20] and mortinatality [21]. Therefore, canine mastitis should be considered a major emergency and must be diagnosed and treated quickly and appropriately [20,22,23,24].

However, knowledge related to its etiology is heterogeneous. Therefore, this study aimed to describe the prevalence of aerobic bacteria in bitch milk, according to the clinical status of the mammary gland, the lactation period, the reproductive status, seasonality, and sheltering in order to contribute to the knowledge that would permit better characterization of the disease and therefore would help in the decision-taking concerning diagnosis and proper treatment.

## 2. Materials and Methods

### 2.1. Animals

Eighty-seven (i.e., 87) bitches between 12 and 168 months of age (SD +/−44.86) with a body weight (BW) between 3 and 65 kg (SD +/−30 kg) were included in the present study. Forty-one dogs (*n* = 41; 47%) were healthy lactating bitches, while 17 (19.5%) were diagnosed with subclinical and 29 (33.3%) with clinical mastitis. All dogs were brought to the Department and Clinic of Reproduction, Obstetrics and Gynaecology, University of Agricultural Sciences and Veterinary Medicine, Cluj-Napoca, Romania, for a gynecology consultation between January 2013 and December 2014. Some of the dogs were included in previously published papers that investigated bitch hematological changes [25], milk and serum CRP levels [26] and milk cytology diagnostics [27] during mastitis episodes.

The dogs were of 29 different breeds, namely German Shepherd (*n* = 18), Cane Corso (*n* = 9), mongrel (*n* = 6), Rottweiler (*n* = 7), Caucasian Shepherd Dog (*n* = 6), Dobermann (*n* = 4), Yorkshire Terrier (*n* = 4), Bichon (*n* = 3), Siberian Husky (*n* = 3), American Bulldog (*n* = 2), Beagle (*n* = 2), English Bulldog (*n* = 2), French Bulldog (*n* = 2), German Shorthaired Pointer (*n* = 2), Pekingese (*n* = 2), Saint Bernard (*n* = 2) and one of each of the following breeds: American Staffordshire Terrier, Bucovina Shepherd Dog, Cocker Spaniel, Basset Hound, Belgian Shepherd, Boxer, Central Asian Shepherd Dog, Dachshund, Golden Retriever, Labrador Retriever, Neapolitan Mastiff, Shih Tzu and Vizsla.

A total of 50.6% were multiparous, 35.6% were primiparous and 10.3% were intact females, while for the remaining 3.4%, no such data were available. Three (*n* = 3; 3.4%) bitches were in the ante-partum period, 70 (80.5%) were in the post-partum period and 14 (16.1%) had *Lactatio sine graviditate*. Detailed individual data are presented in Supplementary Tables S1 and S2.

Subclinical cases of mastitis (*n* = 17; 19.5%) were diagnosed based on clinical evaluation (i.e., lack of any mammary inflammatory signs) and laboratory assays, i.e., presence of an alkaline milk pH (>7) and an increase in both milk (>5 μg/mL) and serum (>10 μg/mL) CRP [26], with the presence of inflammatory cells and bacteria phagocytized on milk smears [27], alongside the presence of bacteria on cultured samples.

Clinical cases of mastitis (*n* = 29; 33.3%), including mammary congestion, galactostasis, *Mastitis acuta* and *Mastitis gangrenosa*, were diagnosed based on clinical evaluation and laboratory changes of overt mastitis, i.e., restlessness; red, hot, engorged and painful mammary glands, with or without modified mammary gland secretion and general signs of illness such as anorexia, fever and tacky mucous membranes with a delayed capillary refill time (CRT) or even septicemia alongside avoiding puppy nursing or failure of puppies to thrive; the presence of an alkaline milk pH (>7) and an increase in both milk (>5 μg/mL) and serum (>10 μg/mL) CRP [26], with the presence of inflammatory cells and bacteria phagocytized on milk smears [27] alongside the presence of bacteria on milk samples.

Bitches with mammary congestion (*n* = 3) presented engorged, hot and painful mammae, with an acidic milk pH (<6.5), with elevated levels of both milk (>5 μg/mL) and serum (>10 μg/mL) CRP [26], but with a lack of any inflammatory cells on milk smears [27].

Bitches with galactostasis (*n* = 13) presented engorged, hot and painful mammae, with or without modified milk secretion, with an alkaline milk pH (7–9.5), and with high levels of both milk (>5 μg/mL) and serum (>10μg/mL) CRP [26] with the presence of degenerated neutrophils, numerous eosinophils and foamy cells with bacteria phagocytized on milk smears [27].

Bitches with *Mastitis acuta* (*n* = 12) presented hot, red, engorged or hardened painful mammary glands, with or without modified milk secretion, with a modified general status, anorexia, fever and tacky mucous membranes and with a delayed CRT. Hematology showed the presence of neutrophilia with a left shift [25]. Milk samples showed the presence of an alkaline milk pH (>7), alongside an elevated milk (>5 μg/mL) and serum (>10 μg/mL) CRP level [26], and the presence of degenerated neutrophils, foamy cells, bacteria, cellular debris and bacteria phagocytized on milk smears [27].

In the bitch with *Mastitis gangrenosa* (*n* = 1), the affected mammary gland presented a dark to purple color and was hot, engorged and painful, with a modified general status, presenting fever, dehydration, anorexia, depression and sepsis. Blood analyses showed the presence of anemia, leukocytosis and thrombocytopenia [25], alongside an alkaline milk sample (>7) with both elevated milk (8.0 μg/mL) and serum (113.4 μg/mL) CRP levels [26] and the presence of neutrophils, foamy cells, bacteria, cellular debris and bacteria phagocytized on the milk smear [27].

### 2.2. Milk Samples

A total of 162 milk samples were collected from the 87 lactating bitches (in the majority of the cases, 2 samples from two different mammary glands were collected from the same animal): healthy (*n* = 41; 76 milk samples) and with subclinical (*n* = 17; 34 milk samples) and clinical (*n* = 29; 52 milk samples) mastitis. After thorough disinfection of the mammary glands with soap, water and alcohol, milk samples were manually obtained and harvested in sterile vials (NuncTM, Waltham, MA, USA). Milk pH and cytology were assessed in fresh samples [27,28,29]. The resting milk samples were used for microbiological analyses.

### 2.3. Microbiological Analyses

For microbiological testing, standard methods were used [30]. In brief, to isolate and identify aerobic bacteria from milk samples, after a 24 h incubation in brain–heart infusion broth (Oxoid Limited, Basingstoke, Hampshire, UK) at 37 °C, samples were cultured on Chapman (Oxoid Limited, Basingstoke, Hampshire, UK) medium for staphylococcal growth, on McConkey (Oxoid Limited, Basingstoke, Hampshire, UK) medium for Enterobacteriaceae, and other Gram-negative organisms and on blood agar (Oxoid Limited, Basingstoke, Hampshire, UK) for Streptococcus species. After isolation, bacterial isolates were identified using Vitek2 (BioMérieux, l’Étoile, France) technology, respecting the manufacturer’s guidelines. Moreover, for *S. pseudintermedius* and *S. intermedius* only, the molecular identification was conducted according to the molecular protocol described in 2010 by Sasaki [31].

### 2.4. Statistical Analysis

Anamnestic data, clinical outcome and microbiological results were stored in Excel. Data were analyzed with GraphPad Prism version 8.0.0 for Windows (GraphPad Software, San Diego, CA, USA, www.graphpad.com, accessed on 12 May 2021). A chi-squared test was used to assess statistical differences between prevalence. A *p-value* less than 0.05 was considered significant.

## 3. Results

### 3.1. Clinical Findings

The detailed clinical data of all the patients included in this study and divided according to clinical signs of mammary gland infection are shown in Supplementary Table S2. Finally, from all the bitches included in the study (17 bitches with subclinical mastitis, 29 bitches with clinical mastitis and 41 healthy bitches), 162 milk samples were collected and assessed.

### 3.2. Microbiological Description of Milk Samples

Among the 162 milk samples included, 93.2% (151/162) resulted in a positive microbiological examination and 6.8% (11/162) were sterile, out of which 63.3% (7/11) were from bitches with *Lactatio sine graviditate*. From the positive samples, 48.34% (73/151) showed Gram-positive bacteria (G+) and 11.26% (17/151) Gram-negative (G−), while from 40.40% (61/151) both Gram+/− species were cultivated. Up to 47 different isolated species were identified; the highest prevalence was observed for *Escherichia coli* (29.1%), *Enterococcus faecium* (11.9%), *Proteus mirabilis* (9.3%), *S. pseudintermedius* (8.6%) and *S. simulans* (6.6%) (Table 1).

Bacterial associations were detected in 61/151 samples. Co-isolation of *Staphylococcus* sp. and *E. coli* was detected in 29.5% of cases, followed by associations between *Staphylococcus* sp. and strains from the Pseudomonadaceae family (4.9%), *Staphylococcus* sp. and *Proteus* sp. in 14.75% of cases, and *Enterococcus* sp. and *E*. *coli* in 6.6%. The other types of associations were identified in a proportion of less than 3%.

From bitches with healthy *mammae*, the highest prevalence was encountered for the Staphylococcaceae family (43.1%), followed by the Enterobacteriaceae (23.8%) and Enterococcaceae families (10.1%) (Table 2).

From bitches with subclinical mastitis, the highest prevalence was identified for the Staphylococcaceae family (36.4%), followed by the Enterobacteriaceae (18.2%), Rhizobiaceae (7.6%), Bacillaceae (7.6%) and Enterococcaceae families (7.6%) (Table 2). Finally, from bitches with clinical mastitis, the highest prevalence was recorded for the Staphylococcaceae family (45.6%), followed by the Enterobacteriaceae (13.2%) and Enterococcaceae families (10.3%) (Table 2).

According to the mammary gland clinical status, the present research shows that the most common isolates are from the Staphylococcaceae and Enterobacteriaceae families, showing a relatively equal distribution among the milk samples collected from the healthy bitches and those with clinical and subclinical mastitis. The bacterial families with the highest prevalence from the samples of healthy and clinically affected mammary glands appear to be the same (Table 2).

In the post-partum period, the highest prevalence was encountered for the Staphylococcaceae family (39.4%), followed by the Enterobacteriaceae (20.7%) and Enterococcaceae families (10.8%) (Table 3).

For bitches with *Lactatio sine graviditate*, the highest prevalence was recorded for the Staphylococcaceae family (60%), followed by the Bacillaceae (15%), Aeromonadaceae (10%) and Streptococcaceae families (10%) (Table 3). In the ante-partum period, the highest prevalence was recorded for the Staphylococcaceae family (43.7%), followed by the Enterobacteriaceae (18.7%), Bacillaceae (12.5%) and Moraxellaceae (12.5%) families (Table 3).

During the different lactation periods, after comparing post-partum females and females with *Lactatio sine graviditate*, a statistical difference was observed for Bacillaceae, Leuconostocaceae, Aeromonadaceae and Enterobacteriaceae families. The Moraxellaceae were statistically different after comparing samples of the post-partum females with the samples from the ante-partum period (Table 3).

From intact bitches, the highest prevalence was recorded for the Staphylococcaceae family (72.7%), followed by the Bacillaceae (18.2%) and Leuconostocaceae families (9.1%) (Table 4). From multiparous bitches, the highest prevalence was recorded for the Staphylococcaceae family (37.5%), followed by the Enterobacteriaceae (24.3%) and Enterococcaceae (10.3%) families (Table 4).

Finally, from primiparous bitches, the highest prevalence was recorded for the Staphylococcaceae family (45.3%), followed by the Enterobacteriaceae (16.3%) and Morganellaceae (12.8%) families (Table 4).

According to the reproductive status, a statistical difference was identified for the Staphylococcaceae and Morganellaceae families’ prevalence in the samples from the multiparous females when compared with the samples from intact and primiparous females, respectively (Table 4).

During the autumn season (September–November), the highest prevalence was registered for the Staphylococcaceae family (42.4%), followed by the Enterobacteriaceae (15.1%), Rhizobiaceae (12.1%) and Pseudomonadaceae families (12.1%) (Table 5).

During the spring season (March–May), the highest prevalence was encountered for the Staphylococcaceae family (39.7%), followed by the Enterobacteriaceae (22.2%) and Enterococcaceae (17.5%) families (Table 5). During the summer (June–August), the highest prevalence was registered for the Staphylococcaceae family (41.3%), followed by the Enterobacteriaceae (25.4%), Bacillaceae (11.1%) and Morganellaceae (11.1%) families (Table 5). Finally, during the winter season (December–February), the highest prevalence was registered for the Staphylococcaceae family (47.9%), followed by the Enterobacteriaceae (14.6%), Enterococcaceae (8.3%) and Morganellaceae (8.3%) families (Table 5).

Moreover, a statistical difference was identified for the Rhizobiaceae family’s prevalence when comparing samples from the autumn with the samples from the other seasons; for the Bacillaceae family’s prevalence, when samples from the autumn were compared with the samples from summer; for the Enterococcaceae family’s prevalence when samples were compared between spring and summer; and for the Pseudomonadaceae family’s prevalence when samples from autumn were compared with samples from spring and summer (Table 5).

According to the sheltering, for the kennel population, the highest prevalence was encountered for the Staphylococcaceae family (39.8%), followed by the Enterobacteriaceae (20.5%) and Enterococcaceae (16.9%) families (Table 6). For the population living inside the house, the highest prevalence was encountered for the Staphylococcaceae family (35.5%), followed by the Enterobacteriaceae (19.3%) and Morganellaceae (16.1%) families (Table 6). For the population housed in the backyard, the highest prevalence was encountered for the Staphylococcaceae family (37.2%), followed by the Enterobacteriaceae (20.2%) and Bacillaceae (8.5%) families (Table 6). For individuals both inside the house and in the backyard, the highest prevalence was encountered for the Staphylococcaceae family (64.7%), followed by the Enterobacteriaceae (17.6%), Bacillaceae (5.9%) and Rhizobiaceae (5.9%) families. Finally, for roaming dogs, the highest prevalence was encountered for the Staphylococcaceae (50%) and Enterococcaceae (50%) families.

Moreover, a statistical difference was identified for the Enterococcaceae family’s prevalence when comparing samples from kennel dogs with the samples from backyard dogs; for the Staphylococcaceae family’s prevalence when samples from kennel dogs were compared with samples from house and backyard dogs; for Caulobacteraceae, the Bacillaceae and Moraxellaceae family’s prevalence when samples from kennel dogs were compared with the samples from street dogs. A statistical difference was also noticed for the Staphylococcaceae and Morganellaceae family when samples from dogs living inside houses were compared with samples from dogs housed both inside and in the backyard and for the Staphylococcaceae family’s prevalence from samples from dogs living in the backyard when compared with samples from dogs living inside and in the backyard. Finally, a statistical difference was also noticed for the Leuconostocaceae, Sporolactobacillaceae, Streptococcaceae, Neisseraceae and Yersiniaceae family prevalences, when samples from dogs housed in backyards were compared with samples from street dogs (Table 6). 

### 3.3. Additional Results

We also analyzed (using the same workflow described in the Materials and Methods section) 24 milk samples derived from 14 bitches without a diagnostic of the mammary gland (due to incomplete laboratory testing) (Supplementary Table S3). These samples (and the relative microbiological results) were excluded from statistical analysis. However, some interesting bacterial species were found, such as *S. lentus, S. epidermidis, Enterococcus villorum, Kocuria rosea, Shewanella putrefaciens* and *Aeromonas hydrophila*. Except for *S. epidermidis*, to the authors’ best knowledge, these strains are firstly described here.

## 4. Discussion

In this report, 34 strains isolated from canine milk are reported for the first time (Table 1; Supplementary Table S3). The majority of strains were present in healthy and diseased bitches, while *A. salmonicida*, *Bacillus lentus*, *S. chromogenes, Streptococcus anginosus* and *S. sanguinis* were isolated only from the milk of bitches with *Lactatio sine graviditate* (Table 1; Supplementary Table S3).

Bitch milk isolates such as *E. faecalis*, *E. faecium*, *Leuconostoc mesenteroides*, *S. aureus*, *S. epidermidis*, *S. hominis* ssp. *hominis*, *S. xylosus*, *S. haemolyticus*, *Micrococcus luteus*, *E. coli* and *Pseudomonas aeruginosa* and other types of bacterial strains such as *Gemella*, *Kocuria*, *Acinetobacter*, *Burkholderia* and *Klebsiella* are also part of the healthy human milk microbiota [32]. In the milk of lactating bitches, the presence of these bacteria may influence the immunological maturation of the mammary gland or prevent the onset of mastitis episodes.

Another salient finding was the isolation of different bacterial species in periparturient bitches compared with *Lactatio sine graviditate* females. Therefore, it is possible that, due to human manipulation of the lactating mammary glands, many of the different isolated strains only from *Lactatio sine graviditate* females, such as *S. sanguinis, S. anginosus* and *S. hominis* ssp. *hominis*, could derive from human skin [33,34,35].

Even though bacterial aerobic isolates from milk of both periparturient and *Lactatio sine graviditate* bitches had been reported before, there is no clear information regarding the possible difference in the relative abundance of cultivable bacteria populations among the two different lactation periods [36]. Since *Lactatio sine graviditate* is a physiological atavism manifestation in the bitch [14,37], the majority (i.e., 65%) of the females develop a nonseptic mastitis (galactostasis) [14,27]. In the current study, the presence of galactostasis (i.e., 13 cases) and the lack of human manipulation could explain why the majority (i.e., 7/11; 63.3%) of the sterile samples were registered in bitches with *Lactatio sine graviditate*. Current data show that *Acinetobacter anitratus, Bacillus sp., E. durans, E. coli, P. stutzeri, Shigella sp., S. epidermidis, S. haemolyticus, S. intermedius, S. simulans* and *Streptococcus* sp. [21,38], alongside many other bacterial species such as *Haemophillus, Moraxella, Staphylococcus*, *Streptococcus* and Lactobacilli [38,39], are part of the resident milk microflora in the healthy mammary glands of lactating bitches. Our results are partially consistent with these findings since *S. intermedius* showed an important etiopathogenic role in the infection of the mammary glands (Table 1).

In bitches, mastitis holds a prevalence of less than 1% of all reported afflictions [40] and less than 6% of all the reproductive pathologies [41]. However, about 13% of post-partum females develop clinical mastitis, with a higher incidence in bitches that whelp large litters or develop mammary congestion [42].

Association between *Staphylococcus* sp. and *E*. *coli* strains and between the *Staphylococcus* sp. and *Pseudomonadaceae* family, alongside *Staphylococcus* sp. and *Proteus* sp., is consistent with that mentioned in the literature [21]. In the current study, we also identified co-isolation of *Enterococcus* sp. with *E. coli* strains.

In the present study, the isolation of *E. coli* and *S. intermedius* strains from the milk of bitches with clinical and subclinical mastitis highlights the critical pathogenic role (also found in the literature) of these pathogens. In an older study [43], *S*. *intermedius*, alongside *S. canis* and *E. coli* strains, was isolated in 66% of the tested milk samples [44], highlighting the septicemic potential that these pathogens might have on nurslings [45], while experimental infections stressed the essential role that *S. intermedius* can play in the pathogenesis of mastitis in lactating bitches [29].

Bacterial strains responsible for puppy mortality, which represent the second most common cause of morbidity and mortality [46], were also found in our study, stressing the possibility of translocating milk pathogens from the bitch to the nurslings. 

With a prevalence of 8.61%, *S. pseudintermedius* strains, which are frequently held responsible for various infections in dogs [47,48,49], were also isolated in the present study, but mainly from healthy mammary glands (Table 1).

*Streptococcus* sp. strains are among the most important opportunistic pathogens in dogs [50]. In our study, this pathogen was isolated mainly from healthy mammary glands, while *S. anginosus* and *S. sanguinis* were recovered from a bitch with *Lactatio sine graviditate* diagnosed with clinical mastitis (Table 1). 

Bacterial strains, which are responsible for nosocomial infections in humans [35,51,52,53,54,55,56,57,58,59,60], such as *Agrobacterium radiobacter*, *Burkholderia cepacia*, *Chromobacterium violaceum*, *Ochrobactrum anthropi*, *P. luteola*, *S. angiosus*, *S. capitis*, *S. cohni cohni*, *S. haemolyticus*, *S. hominis* ssp. *hominis* and *S. warneri*, were also isolated from the milk of the lactating bitches in the current study (Table 2). This finding can highlight the importance of a One Health approach in diagnosing and treating common infections in dogs since they can act as a reservoir for potentially zoonotic diseases. It could also drive a decision-taking approach and operative protocols to contain spillover while also reducing the use of antibiotics.

Due to close contact between humans and dogs, the transmission of pathogenic strains from one to another is highly possible [61]. In addition, cases of human infections due to *E. coli* [17] and *S. canis* [50,62] from pets have been reported.

The isolation of some zoonotic strains from the milk of lactating bitches highlights the important role of maintaining a clean and healthy mammary gland for both the litter and human health. In addition, these strains found in bitch milk should emphasize to breeders, owners and veterinary staff the possibility of hazardous pathogen transfer between them and lactating bitches.

Kennel, shelter and litter hygiene needs to be properly addressed alongside a low manipulation of the lactating mammae where possible. To minimize possible infections throughout manual contact, regardless of the lactation period, it is highly recommended to limit mammary gland contact as much as possible alongside providing a healthy and clean environment [14,17].

Ignoring mastitis episodes in bitches, especially subclinical ones, will enhance mammary complications, such as the development of mammary duct ectasia [63] or *Mastitis gangrenosa* with septicemia, with a poor prognosis for both the bitch and the pups [18,64].

As shown in human medicine [65,66], further research should stress whether the presence of continuous nursing in lactating bitches and suckling from pups, from birth to weaning, can prevent (in both litter and bitches) the rise of different afflictions such as mortinatality, gastrointestinal infections, otitis, atopic dermatitis, obesity, type 2 diabetes and ovarian and mammary cancer.

Even though milk samples were manually collected, respecting all the hygienic protocols, the anatomical particularities of the bitch mammary glands and the lack of molecular identification of the isolated bacterial strains represent the main limitations of the current study.

Future research should also concentrate on identifying the prevalence of anaerobic bacterial load of the milk secretion alongside the correlation between environmental bacterial pathogens and their influence on the bacterial milk load in lactating bitches.

## 5. Conclusions

The prevalence of bacterial strains can be influenced by the lactating period, lactation season, gestation type and sheltering conditions. From *Lactatio sine graviditate* bitches, the milk samples collected seem to have a lower bacteriological pressure than the periparturient females since more than half of the samples are sterile.

In the post-partum period, for multiparous bitches, in the spring and winter seasons and according to the mammary gland clinical status, the most common cultivable bacteria are the Staphylococcaceae, Enterobacteriaceae and Enterococcaceae families.

In the ante-partum period, for intact bitches or in females with *Lactatio sine graviditate*, the most common cultivable bacteria at the family level by relative abundance are the Staphylococcaceae and Bacillaceae families, followed by the Moraxellaceae, Leuconostocaceae and Aeromonadaceae families.

For primiparous bitches, in the autumn and summer seasons, the most common cultivable bacteria at the family level by relative abundance are the Staphylococcaceae and Enterobacteriaceae families, followed by the Morganellaceae, Rhizobiaceae and Bacillaceae families.

With the exception of bitches that were sheltered inside, all other types of sheltering influenced the majority of the bacterial families’ prevalence.

## Figures and Tables

**Table 1:** Bacterial species isolated from milk samples. Confidence intervals at 95% (CI) were calculated using the formula for prevalence.

Families N; %/CI%	Pathogen	N; %	CI%	AP	PP	LSG	H	Sb	C
Moraxacellaceae 4; 2.65/6.22–16.30	Acinetobacter iwoffii *	2; 1.3	unv–3.15		2		2		
	Acinetobacter ursingii *	2; 1.3	unv–3.15	2			2		
Aerococcaceae 1; 0.66/unv–1.96	Aerococcus viridans *	1; 0.7	unv–1.96		1		1		
Aeromonadaceae 2; 1.32/unv–3.15	Aeromonas salmonicida *	2; 1.3	unv–3.15			2			2
Rhizobiaceae 8; 5.3/1.73–8.87	Agrobacterium radiobacter *	8; 5.	1.73–8.87		8		3	5	
Bacillaceae 14; 9.27/4.65–13.90	*Bacillus cereus*	2; 1.3	unv–3.15		2		2		
	Bacillus pumilus *	4; 2.6	0.09–5.21	1		3		1	3
	*Bacillus* sp.	5; 3.3	0.46–6.17	1	4		1	1	3
	Bacillus subtilis *	1; 0.7	unv–1.96		1			1	
	Brevibacillus laterosporus *	2; 1.3	unv–3.15		2			2	
Caluobacteraceae 1; 0.66/unv–1.96	Brevundimonas vesicularis *	1; 0.7	unv–1.96		1		1		
Burkholderiaceae 1; 0.66/unv–1.96	Burkholderia cepacia *	1; 0.7	unv–1.96		1			1	
Neisseraceae 1; 0.66/unv–1.96	Chromobacterium violaceum *	1; 0.7	unv–1.96		1			1	
Enterococcaceae 22; 14.57/8.94–20.20	Enterococcus avium *	4; 2.6	0.09–5.21		4		1	3	
	*Enterococcus faecium*	18; 11.9	6.75–17.09		18		10	1	7
Enterobacteriaceae 47; 31.13/23.74–38.51	*Escherichia coli*	44; 29.1	21.89–36.39	3	41		25	10	9
	*Klebsiella* sp.	2; 1.3	unv–3.15		2		1	1	
	*Enterobacter cloacae* complex *	1; 0.7	unv–1.96		1			1	
Sporolactobaillaceae 1; 0.66/unv–1.96	Gemella bergeri *	1; 0.7	unv–1.96		1		1		
Leuconostocaceae 1; 0.66/unv–1.96	*Leuconostoc mesenteroides* ssp. cremoris *	1; 0.7	unv–1.96			1			1
Morganellaceae 17; 11.26/6.22–16.3	*Morganella morganii* ssp. morganii *	1; 0.7	unv–1.96		1			1	
	*Proteus mirabilis*	14; 9.3	4.65–13.9	1	13		5	5	4
	Proteus vulgaris *	2; 1.3	unv–3.15		2				2
Brucellaceae 2; 1.32/unv–3.15	Ochrobactrum anthropi *	2; 1.3	unv–3.15		2		1	1	
Pseudomonadaceae 10; 6.62/2.66–10.59	*Pseudomonas aeruginosa*	7; 4.6	1.28–7.99		7			5	2
	Pseudomonas luteola *	2; 1.3	unv–3.15		2		2		
	Pseudomonas marginata *	1; 0.7	unv–1.96	1					1
Staphylococcaceae 99; 65.56/57.98–73.14	*Micrococcus luteus*	5; 3.3	0.46–6.17		5		1	3	1
	*Staphylococcus aureus*	1; 0.7	unv–1.96		1				1
	Staphylococcus capitis *	1; 0.7	unv–1.96		1		1		
	Staphylococcus chromogenes *	2; 1.3	unv–3.15			2			2
	*Staphylococcus cohnii* ssp. cohni *	1; 0.7	unv–1.96		1				1
	*Staphylococcus haemolyticus*	4; 2.6	0.09–5.21	1	3		3	1	
	*Staphylococcus hominis* ssp. hominis *	6; 4	0.86–7.09		1	5	1	1	4
	*Staphylococcus hyicus*	1; 0.7	unv–1.96		1				1
	*Staphylococcus intermedius*	5; 3.3	0.46–6.17	1	4				5
	*Staphylococcus pseudintermedius*	13; 8.6	4.14–13.08		12	1	9		4
	Staphylococcus sciuri *	1; 0.7	unv–1.96		1		1		
	*Staphylococcus simulans*	10; 6.6	2.66–10.59	1	9		5	4	1
	*Staphylococcus* sp.	39; 25.8	18.85–32.81	4	31	4	23	7	9
	Staphylococcus vitulis *	1; 0.7	unv–1.96		1		1		
	*Staphylococcus warneri*	2; 1.3	unv–3.15		2			2	
	Staphylococcus xylosus *	7; 4.6	1.28–7.99		7		1	6	
Streptococcaceae 7; 4.64/1.28–7.99	Streptococcus anginosus *	1; 0.7	unv–1.96			1			1
	*Streptococcus sanguinis*	1; 0.7	unv–1.96			1			1
	*Streptococcus* sp.	5; 3.3	0.46–6.17		5		4	1	
Yersiniaceae 1; 0.66/unv–1.96	Yersinia enterocolitica *	1; 0.7	unv–1.96		1				1

* Milk bacterial species described for the first time in bitch milk; PP = post-partum; LSG = *Lactatio sine graviditate*; AP = ante-partum; H = healthy; Sb = subclinical mastitis; C = clinical mastitis; N = number of observation; CI = confidence interval; unv = unavailable (due to negative value, CI was not indicated).

**Table 2:** Relative abundance (%) of cultivable bacteria at the family level according to mammary gland clinical status.

	H (109)	Sb (66)	C (68)	H vs. Sb	Sb vs. C	H vs. C
Brucellaceae	0.9	1.5	0	ns	ns	ns
Caulobacteraceae	0.9	0	0	ns	ns	ns
Rhizobiaceae	2.7	7.6	0	ns	ns	ns
Aerococcaceae	0.9	0	0	ns	ns	ns
Bacillaceae	2.7	7.6	8.8	ns	ns	ns
Enterococcaceae	10.1	7.6	10.3	ns	ns	ns
Leuconostocaceae	0	0	1.5	ns	ns	ns
Sporolactobacillaceae	0.9	0	0	ns	ns	ns
Staphylococcaceae	43.1	36.4	45.6	ns	ns	ns
Streptococcaceae	3.7	1.5	2.9	ns	ns	ns
Burkholderiaceae	0	3	0	ns	ns	ns
Neisseraceae	0	1.5	0	ns	ns	ns
Aeromonadaceae	0	0.	2.9	ns	ns	ns
Enterobacteriaceae	23.8	18.2	13.2	ns	ns	ns
Moraxellaceae	3.7	0	0	ns	ns	ns
Morganellaceae	4.6	7.6	8.8	ns	ns	ns
Pseudomonadaceae	1.8	7.6	4.4	ns	ns	ns
Yersiniaceae	0	0	1.5	ns	ns	ns

H = healthy; Sb = subclinical mastitis; C = clinical mastitis; ns = not significant.

**Table 3:** Relative abundance (%) of cultivable bacteria at the family level according to lactation period.

	PP (213)	LSG (20)	AP (16)	PP vs. LSG	LSG vs. AP	PP vs. AP
Brucellaceae	0.9	0	0	ns	ns	ns
Caulobacteraceae	0.5	0	0	ns	ns	ns
Rhizobiaceae	3.8	0	0	ns	ns	ns
Aerococcaceae	0.5	0	0	ns	ns	ns
Bacillaceae	4.2	15	12.5	0.0371	ns	ns
Enterococcaceae	10.8	0	0	ns	ns	ns
Leuconostocaceae	0	5	0	0.011	ns	ns
Sporolactobacillaceae	0.5	0	0	ns	ns	ns
Staphylococcaceae	39.4	60	43.7	ns	ns	ns
Streptococcaceae	2.3	10	0	ns	ns	ns
Burkholderiaceae	0.9	0	0	ns	ns	ns
Neisseraceae	0.5	0	0	ns	ns	ns
Aeromonadaceae	0	10	0	<0.0001	ns	ns
Enterobacteriaceae	20.7	0	18.7	0.024	ns	ns
Moraxellaceae	0.9	0	12.5	ns	ns	0.0007
Morganellaceae	7.5	0	6.2	ns	ns	ns
Pseudomonadaceae	6.1	0	6.2	ns	ns	ns
Yersiniaceae	0.5	0	0	ns	ns	ns

PP = post-partum; LSG = *Lactatio sine graviditate*; AP = ante-partum; ns = not significant.

**Table 4:** Relative abundance (%) of cultivable bacteria at the family level according to reproductive status.

	I (11)	M (136)	P (86)	I vs. M	M vs. P	I vs. P
Brucellaceae	0	1.5	0	ns	ns	ns
Caulobacteraceae	0	0.7	0	ns	ns	ns
Rhizobiaceae	0	3.7	3.5	ns	ns	ns
Aerococcaceae	0	0.7	0	ns	ns	ns
Bacillaceae	18.2	5.9	4.6	ns	ns	ns
Enterococcaceae	0	10.3	8.1	ns	ns	ns
Leuconostocaceae	9.1	0	0	ns	ns	ns
Sporolactobacillaceae	0	0.7	0	ns	ns	ns
Staphylococcaceae	72.7	37.5	45.3	0.0485	ns	ns
Streptococcaceae	0	2.2	2.3	ns	ns	ns
Burkholderiaceae	0	1.5	0	ns	ns	ns
Neisseraceae	0	0	1.2	ns	ns	ns
Aeromonadaceae	0	0	0	ns	ns	ns
Enterobacteriaceae	0	24.3	16.3	ns	ns	ns
Moraxellaceae	0	2.9	0	ns	ns	ns
Morganellaceae	0	3.7	12.8	ns	0.0105	ns
Pseudomonadaceae	0	3.7	5.8	ns	ns	ns
Yersiniaceae	0	0.7	0	ns	ns	ns

I = intact; M = multiparous; P = primiparous; ns = not significant.

**Table 5:** Relative abundance (%) of cultivable bacteria at the family level according to the season of sampling. A chi-squared test was used to compare prevalence between groups.

	Sp (63)	Sm (63)	A (66)	W (48)	Sp vs. Sm	Sp vs. W	A vs. Sp	A vs. Sm	A vs. W	Sm vs. W
Brucellaceae	0	0	3	0	ns	ns	ns	ns	ns	ns
Caulobacteraceae	0	0	1.5	0	ns	ns	ns	ns	ns	ns
Rhizobiaceae	0	0	12.1	0	ns	ns	0.0339	0.0128	0.0332	ns
Aerococcaceae	0	0	0	2.1	ns	ns	ns	ns	ns	ns
Bacillaceae	7.9	11.1	0	4.2	ns	ns	ns	0.0166	ns	ns
Enterococcaceae	17.5	4.8	6.1	8.3	0.0472	ns	ns	ns	ns	ns
Leuconostocaceae	0	0	0	2.1	ns	ns	ns	ns	ns	ns
Sporolactobacillaceae	1.6	0	0	0	ns	ns	ns	ns	ns	ns
Staphylococcaceae	39.7	41.3	42.4	47.9	ns	ns	ns	ns	ns	ns
Streptococcaceae	3.2	0	1.5	4.2	ns	ns	ns	ns	ns	ns
Burkholderiaceae	0	3.2	0	0	ns	ns	ns	ns	ns	ns
Neisseraceae	0	0	0	2.1	ns	ns	ns	ns	ns	ns
Aeromonadaceae	0	3.2	0	0	ns	ns	ns	ns	ns	ns
Enterobacteriaceae	22.2	25.4	15.1	14.6	ns	ns	ns	ns	ns	ns
Moraxellaceae	1.59	0	3	2.1	ns	ns	ns	ns	ns	ns
Morganellaceae	4.76	11.1	3	8.3	ns	ns	ns	ns	ns	ns
Pseudomonadaceae	1.59	0	12.1	2.1	ns	ns	0.0453	0.0128	ns	ns
Yersiniaceae	0	0	0.00	2.1	ns	ns	ns	ns	ns	ns

Sp = spring; Sm = summer; A = autumn; W = winter; ns = not significant.

**Table 6:** Relative abundance (%) of cultivable bacteria at the family level according to sheltering. A chi-squared test was used to compare prevalence between groups.

	K (83)	H (31)	B (94)	HB (34)	S (2)	K vs. H	K vs. B	K vs. HB	K vs. S	H vs. B	H vs. HB	H vs. S	B vs. HB	B vs. S
Brucellaceae	0	0	2.1	0	0	ns	ns	ns	ns	ns	ns	ns	ns	ns
Caulobacteraceae	1.2	0	0	0	0	ns	ns	ns	0.0016	ns	ns	ns	ns	ns
Rhizobiaceae	3.6	0	3.2	5.9	0	ns	ns	ns	ns	ns	ns	ns	ns	ns
Aerococcaceae	0	3.2	0	0	0	ns	ns	ns	ns	ns	ns	ns	ns	ns
Bacillaceae	1.2	9.7	8.5	5.9	0	ns	ns	ns	0.0016	ns	ns	ns	ns	ns
Enterococcaceae	16.9	6.4	5.3	2.9	50	ns	0.0255	ns	ns	ns	ns	ns	ns	ns
Leuconostocaceae	0	0	1.1	0	0	ns	ns	ns	ns	ns	ns	ns	ns	0.0007
Sporolactobacillaceae	0	0	1.1	0	0	ns	ns	ns	ns	ns	ns	ns	ns	0.0007
Staphylococcaceae	39.8	35.5	37.2	64.7	50	ns	ns	0.0244	ns	ns	0.0353	ns	0.0104	ns
Streptococcaceae	3.6	6.4	1.1	2.9	0	ns	ns	ns	ns	ns	ns	ns	ns	0.0007
Burkholderiaceae	0	0	2.1	0	0	ns	ns	ns	ns	ns	ns	ns	ns	ns
Neisseraceae	0	0	1.1	0	0	ns	ns	ns	ns	ns	ns	ns	ns	0.0007
Aeromonadaceae	0	0	2.1	0	0	ns	ns	ns	ns	ns	ns	ns	ns	ns
Enterobacteriaceae	20.5	19.3	20.2	17.6	0	ns	ns	ns	ns	ns	ns	ns	ns	ns
Moraxellaceae	2.4	3.2	0	0	0	ns	ns	ns	0.0325	ns	ns	ns	ns	ns
Morganellaceae	7.2	16.1	6.4	0	0	ns	ns	ns	ns	ns	0.0487	ns	ns	ns
Pseudomonadaceae	3.6	0	7.4	0	0	ns	ns	ns	ns	ns	ns	ns	ns	ns
Yersiniaceae	0	0	1.1	0	0	ns	ns	ns	ns	ns	ns	ns	ns	0.0007

Abbreviations: K = kennel; H = house; B = backyard; HB = house and backyard; S = street; ns = not significant.

## Data Availability

Data sharing is not applicable to this article.

## Supplementary Materials

Supplementary materials are available at https://www.mdpi.com/article/10.3390/ani11113259/s1. Table S1: Detailed individual data of the dogs included in the study, Table S2: Anamnestic data related to the dogs included in the study, Table S3: Detailed individual data, with the microbiological diagnostic, of the dogs removed from the study.
