# Supplementary material for: Aerobic Isolates from Gestational and Non-Gestational Lactating Bitches (Canis lupus familiaris)
Source: Animals (Basel). 2021 Nov 14;11(11):3259. doi: 10.3390/ani11113259 (PMC8614458; doi:10.3390/ani11113259)
Supplement: Supplementary file 1 [file animals-11-03259-s001.zip › Supplementary Table S1_rev GM.pdf]

Table S1. Detailed individual data of the dogs included in the study.

	Breed	Years	Months	Body weight	Lactation period	Diagnostic	Reproduction status
1	Rottweiler	3 years	36	45 kg	*PP*	Mastitis acuta	Primiparous
2	Cocker Spaniel	10 years	120	15 kg	*LSG*	Mastitis acuta	Intact
3	Mongrel	5 years	60	30 kg	*PP*	Mastitis acuta	NDA
4	Caucasian Shepherd Dog	4 years	48	55 kg	*PP*	Mastitis acuta	Multiparous
5	Bichon	4 years	48	8 kg	*LSG*	Mastitis acuta	Primiparous
6	Dobermann	1.4 years	16	35 kg	*PP*	Healthy	Primiparous
7	Vizsla	3 years	36	23 kg	*PP*	Mastitis acuta	Primiparous
8	Boxer	6 years	72	26 kg	*PP*	Mastitis acuta	Multiparous
9	Rottweiler	5 years	60	40 kg	*PP*	Subclinical mastitis	Multiparous
10	Mongrel	12 years	144	35 kg	*AP*	Mastitis acuta	Primiparous
11	Caucasian Shepherd Dog	4.5 years	53	65 kg	*PP*	Subclinical mastitis	Multiparous
12	Beagle	6 years	72	16 kg	*PP*	Healthy	Multiparous
13	English Bulldog	3 years	36	25 kg	*PP*	Subclinical mastitis	Multiparous
14	Cane Corso	5 years	60	45 kg	*PP*	Mastitis acuta	Multiparous
15	Basset Hound	2 years	24	30 kg	*PP*	Subclinical mastitis	Primiparous
16	Yorkshire Terrier	2.5 years	29	5 kg	*PP*	Mammary congestion	Primiparous
17	German Shepherd	7 years	84	30 kg	*PP*	Subclinical mastitis	Multiparous
18	German Shepherd	5 years	60	30 kg	*PP*	Healthy	Multiparous
19	German Shepherd	6 years	72	30 kg	*PP*	Healthy	Multiparous
20	English Bulldog	2 years	24	20 kg	*PP*	Healthy	Primiparous
21	Siberian Husky	2.5 years	29	25 kg	*PP*	Healthy	Multiparous
22	Shih Tzu	2 years	24	7 kg	*PP*	Healthy	Primiparous
23	Pekingese	3 years	36	3 kg	*LSG*	Healthy	Intact
24	Mongrel	1.5 years	17	50 kg	*PP*	Subclinical mastitis	Multiparous
25	Rottweiler	3 years	36	40 kg	*PP*	Healthy	Multiparous
26	Caucasian Shepherd Dog	5 years	60	60 kg	*PP*	Subclinical mastitis	Multiparous
27	Saint Bernard	6 years	72	50 kg	*PP*	Healthy	Multiparous
28	Rottweiler	1.3 years	15	25 kg	*PP*	Galactostasis	Primiparous
29	Cane Corso	3 years	36	45 kg	*PP*	Healthy	Primiparous
30	Dobermann	3 years	36	35 kg	*PP*	Galactostasis	Primiparous
31	Bichon	5 years	60	7 kg	*PP*	Subclinical mastitis	Multiparous
32	American Staffordshire Terrier	1.5 years	17	23 kg	*PP*	Healthy	Primiparous
33	Rottweiler	1.5 years	17	40 kg	*LSG*	Galactostasis	Intact
34	German Shepherd	3 years	36	30 kg	*PP*	Healthy	Multiparous
35	German Shepherd	3 years	36	30 kg	*PP*	Healthy	Multiparous
36	Labrador retriever	4 years	48	30 kg	*PP*	Galactostasis	Multiparous
37	Caucasian Shepherd Dog	1.8 years	20	51 kg	*PP*	Subclinical mastitis	Primiparous
38	Neapolitan Mastiff	3 years	36	51 kg	*LSG*	Galactostasis	Intact
39	Cane Corso	4 years	48	40 kg	*PP*	Subclinical mastitis	Multiparous
40	Yorkshire Terrier	1.2 years	14	5 kg	*PP*	Healthy	Primiparous
41	Cane Corso	3 years	36	50 kg	*PP*	Subclinical mastitis	Multiparous
42	Pekingese	2.3 years	27	3 kg	*PP*	Healthy	Primiparous
43	French Bulldog	3 years	36	12 kg	*LSG*	Galactostasis	Intact
44	Cane Corso	3 years	36	40 kg	*PP*	Healthy	Multiparous
45	German Shepherd	3 years	36	30 kg	*PP*	Healthy	Primiparous
46	Dobermann	5 years	60	40 kg	*LSG*	Subclinical mastitis	NDA
47	American Bulldog	3.5 years	41	40 kg	*PP*	Healthy	Multiparous
48	Bichon	14 years	168	8 kg	*LSG*	Galactostasis	Multiparous
49	Rottweiler	2.5 years	29	40 kg	*PP*	Healthy	Primiparous
50	Central Asian Shepherd Dog	7 years	84	50 kg	*PP*	Subclinical mastitis	Multiparous
51	Mongrel	3 years	36	10 kg	*LSG*	Galactostasis	Intact
52	Saint Bernard	3 years	36	40 kg	*PP*	Healthy	Primiparous
53	Rottweiler	5.4 years	64	56 kg	*LSG*	Galactostasis	Multiparous
54	Beagle	3 years	36	16 kg	*PP*	Healthy	Multiparous
55	Mongrel	2 years	24	3 kg	*PP*	Healthy	Primiparous
56	German Shepherd	3 years	36	25 kg	*PP*	Healthy	Multiparous
57	German Shepherd	3.5 years	41	27 kg	*PP*	Healthy	Multiparous
58	German Shepherd	3 years	36	30 kg	*PP*	Healthy	Multiparous
59	German Shepherd	3 years	36	30 kg	*PP*	Healthy	Multiparous
60	German Shepherd	7 years	84	35 kg	*PP*	Healthy	Multiparous
61	Yorkshire Terrier	1.3 years	15	5 kg	*PP*	Mastitis gangrenosa	Primiparous

62	Caucasian Shepherd Dog	5 years	60	50 kg	*AP*	Healthy	Multiparous
63	Yorkshire Terrier	3 years	36	3 kg	*PP*	Healthy	Multiparous
64	American Bulldog	1.3 years	15	35 kg	*LSG*	Galactostasis	Intact
65	German Shepherd	1.5 years	17	20 kg	*PP*	Mammary congestion	Primiparous
66	German Shepherd	6 years	72	25 kg	*PP*	Mammary congestion	Multiparous
67	German Shepherd	2.5 years	29	20 kg	*PP*	Healthy	Multiparous
68	Belgian Shepherd	5.5 years	65	20 kg	*PP*	Healthy	Multiparous
69	Mongrel	4 years	48	20 kg	*PP*	Mastitis acuta	Multiparous
70	Caucasian Shepherd Dog	3 years	36	60 kg	*AP*	Healthy	Multiparous
71	Cane Corso	3 years	36	45 kg	*PP*	Healthy	Primiparous
72	German Shepherd	5 years	60	25 kg	*PP*	Subclinical mastitis	Multiparous
73	German Shepherd	1.5 years	17	25 kg	*PP*	Healthy	Primiparous
74	German Shepherd	1.5 years	17	25 kg	*PP*	Healthy	Primiparous
75	German Shorthaired Pointer	6 years	72	31 kg	*PP*	Healthy	Primiparous
76	Siberian Husky	3 years	36	25 kg	*PP*	Healthy	Multiparous
77	Cane Corso	4 years	48	35 kg	*PP*	Mastitis acuta	Multiparous
78	Bucovina Shepherd Dog	3.3 years	39	44 kg	*LSG*	Galactostasis	NDA
79	German Shepherd	5 years	60	30 kg	*PP*	Healthy	Multiparous
80	Cane Corso	3 years	36	45 kg	*PP*	Subclinical mastitis	Multiparous
81	Cane Corso	3 years	36	40 kg	*PP*	Subclinical mastitis	Primiparous
82	Siberian Husky	4 years	48	12 kg	*LSG*	Galactostasis	Intact
83	French Bulldog	3 years	36	13 kg	*PP*	Healthy	Primiparous
84	Golden Retriever	4 years	48	25 kg	*PP*	Healthy	Primiparous
85	Dachshund	1 year	12	8 kg	*LSG*	Galactostasis	Intact
86	Dobermann	1.8 years	20	40 kg	*PP*	Mastitis acuta	Primiparous
87	German Shorthaired Pointer	5 years	60	20 kg	*PP*	Subclinical mastitis	Primiparous

Abbreviations: LSG—*Lactatio sine graviditate*; AP—*Ante-partum*; PP—*Post-partum*; NDA—No data available.
