# Supplementary material for: Aerobic Isolates from Gestational and Non-Gestational Lactating Bitches (Canis lupus familiaris)
Source: Animals (Basel). 2021 Nov 14;11(11):3259. doi: 10.3390/ani11113259 (PMC8614458; doi:10.3390/ani11113259)
Supplement: Supplementary file 1 [file animals-11-03259-s001.zip › Supplementary Table S2_rev_ GM.pdf]

**Table S2**. Anamnestic data related to the dogs included in the study.

| | | **Healthy N=41** | **Subclinical mastitis N=17** | **Clinical mastitis N=29** |
|---|---|---|---|---|
| Age | | 1.2-7 years | 1.5-7 years | 1-14 years |
| Bodyweight | | 3-60 kg | 7-65 kg | 5-56 kg |
| Season | Spring | 14(34.1%) | 1 (5.9%) | 12 (41.4%) |
| | Summer | 9 (22.0%) | 5 (29.4%) | 7 (24.1%) |
| | Autumn | 10 (24.4%) | 7 (41.2%) | 5 (17.2%) |
| | Winter | 8 (19.5%) | 4 (23.5%) | 5 (17.2%) |
| Type of shelter | Backyard | 10 (24.4%) | 9 (52.9%) | 11 (37.9%) |
| | Flat | 5 (12.2%) | 1 (5.9%) | 5 (17.2%) |
| | House and backyard | 1 (2.4%) | - | 2 (6.9%) |
| | House | 6 (14.06%) | - | 6 (20.7%) |
| | Kennel | 19 (46.3%) | 7 (41.2%) | 4 (13.8%) |
| | Street | | | 1(3.4%) |
| Type of food | Commercial | 17 (41.5%) | 6 (35.3%) | 11 (37.9%) |
| | Commercial/Cooked | 16 (39.0%) | 7 (41.2%) | 8 (27.6%) |
| | Cooked | 4 (9.8%) | 1 (5.9%) | 5 (17.2%) |
| | NDA | 4 (9.8%) | 3 (17.6%) | 5 (17.2%) |
| History | LSG | 1 (2.4%) | 1 (5.9%) | 5 (17.2%) |
| | Obstetrical | 4 (9.8%) | 4 (23.5%) | 3 (10.3%) |
| | Other pathology | 1 (2.4%) | 1 (5.9%) | |
| | NDA | 35 (85.4%) | 11 (64.7%) | 21 (72.4%) |
| Type of coitus | AI | 7 (17.1%) | 4 (23.5%) | 1 (3.4%) |
| | Natural controlled | 29 (70.7%) | 11 (64.7%) | 9 (31.0%) |
| | Natural uncontrolled | 2 (4.9%) | 1(5.9%) | 6 (20.7%) |
| | NDA | 3 (7.5%) | 1 (5.9%) | 13 (44.8%) |
| Reproduction status | Intact | 1 (2.4%) | - | 8 (27.6%) |
| | Primiparous | 17 (41.5%) | 4 (23.5%) | 10 (34.5%) |
| | Multiparous | 23 (56.1%) | 12 (70.6%) | 9 (31.0%) |
| | NDA | - | 1 (5.9%) | 2 (6.9%) |
| Lactation period | LSG | 1 (2.4%) | 1 (5.9%) | 12 (41.4%) |
| | AP | 2 (4.9%) | - | 1 (3.4%) |
| | PP | 38 (92.7%) | 16 (94.1%) | 16 (55.2%) |
| Lactation age in weeks | 1st | 21 (51.2%) | 12 (70.6%) | 8 (27.6%) |
| | 2nd | 9 (22.0%) | 2 (11.8%) | 3 (10.3%) |
| | 3rd | 5 (12.2%) | 1 (5.9%) | 2 (6.9%) |
| | 4th | - | - | - |
| | 5th | 2 (4.9%) | - | - |
| | 7th | 1 (2.4%) | - | 1 (3.4%) |
| | 8th | 1 (2.4%) | 1 (5.9%) | - |
| | NDA | 2 (4.9%) | 1 (5.9%) | 15 (51.7%) |
| Gestation duration | | 51-67 days | 54-64 days | 56-67 days |
| Mammary gland clinical signs | Hard without modified milk secretion | - | - | 7 (24.1%) |
| | Hard with modified milk secretion | - | - | 2 (6.9%) |
| | Hard and sensitive | - | - | 5 (17.2%) |

| | | | | |
|---|---|---|---|---|
| | Modified milk secretion | - | - | 5 (17.2%) |
| | No overt signs | 41 (100.0%) | 17 (100.0%) | 10 (34.5%) |
| Concurrent affections | Mortinatality | 14 (34.1%) | 10 (58.8%) | 4 (13.8%) |
| | Small litter | 3 (7.3%) | - | 2 (6.9%) |
| | Obstetrical | 3 (7.3%) | 4 (23.5%) | 7 (24.1%) |
| | Other pathology | 1 (2.4%) | - | 5 (17.2%) |
| | NDA | 20 (48.8%) | 3 (17.6%) | 11(37.9%) |
| Laboratory assays | | | | |
| Milk pH | Acidic (6.0-6.5) | 25 (61.0%) | 2 (11.8%) | 3 (10.3%) |
| | Alkaline (7.0-9.5) | 14 (34.1%) | 14 (82.4%) | 23 (79.3%) |
| | NDA | 2 (4.9%) | 1 (5.9%) | 3 (10.3%) |
| CRP | Milk | 0.1–2.5μg/mL | 2.9–20.7μg/mL | 2.8–9.2μg/mL |
| | Serum | 3.9–8.0μg/mL | 8.1–67.9μg/mL | 5.2–81.7 μg/mL |
| Milk cytology | | Few somatic and squamous cells | Moderate somatic cells, degenerated neutrophils, foamy and epithelial cells, bacteria and phagocytosis | Increased somatic cells, degenerated neutrophils, eosinophils, foamy cells, cellular debris, erythrocytes, bacteria, and phagocytosis |

Abbreviations: LSG=*Lactatio sine graviditate*; AP=*Ante-partum*; PP=*Post-partum*; AI=Artificial insemination; NDA=No data available; CRP=C-reactive protein; N=number of observations.
