# Supplementary material for: Aerobic Isolates from Gestational and Non-Gestational Lactating Bitches (Canis lupus familiaris)
Source: Animals (Basel). 2021 Nov 14;11(11):3259. doi: 10.3390/ani11113259 (PMC8614458; doi:10.3390/ani11113259)
Supplement: Supplementary file 1 [file animals-11-03259-s001.zip › Supplementary Table S3_rev GM.pdf]

Table S3. Detailed individual data, with the microbiological diagnostic, of the dogs removed from the study.

	Breed	Years	Months	Body weight	Lactation period	Microbiological Diagnostic	Reproduction status
1	Miniature Schnauzer	6 years	72	8 kg	*PP*	*P. mirabilis*	Multiparous
2	Cocker Spaniel	4 years	48	25 kg	*PP*	*S. sciuri*	Multiparous
3	Mongrel	3 years	36	30 kg	*PP*	*E. vilorum/K. rosea*	NDA
4	Siberian Husky	1.5 years	17	20 kg	*PP*	*Staphylococcus* sp.	Multiparous
5	Siberian Husky	1.8 years	20	25 kg	*PP*	*Staphylococcus* sp.	Primiparous
6	American Staffordshire Terrier	1 year	12	22 kg	*LSG*	Sterile	NDA
7	German Shepherd	4.5 years	53	25 kg	*PP*	*A. radiobacter/A. hydrophila*	Multiparous
8	German Shepherd	3.5 years	41	25 kg	*PP*	*S. lentus/S. putrefaciens*	Multiparous
9	Miniature Schnauzer	7 years	84	10 kg	*PP*	*Staphylococcus* sp.	Multiparous
10	Bucovina Shepherd Dog	11 years	132	40 kg	*LSG*	*S. aureus*	NDA
11	Mongrel	5 years	60	10 kg	*PP*	*L. mesenteroides* ssp *cremoris/S. epidermidis*	Multiparous
12	German Shepherd	3.5 years	41	20 kg	*PP*	*B. cereus*	Multiparous
13	Bichon	10 months	10	5 kg	*LSG*	*B. lentus*	Intact

Abbreviations:PP: post-partum; LSG: *Lactatio sine graviditate*; AP: ante-partum; NDA—No data available.
